# Online food delivery services: cross-sectional study of consumers’ attitude in Malaysia during and after the COVID-19 pandemic

**DOI:** 10.12688/f1000research.73014.1

**Published:** 2021-09-27

**Authors:** Sin Yin Tan, Su Yin Lim, Sook Fern Yeo

**Affiliations:** 1 Faculty of Information Science and Technology, Multimedia University, Melaka, 75450, Malaysia; 2 Faculty of Business, Multimedia University, Melaka, 75450, Malaysia

**Keywords:** Online food delivery services, Continuance intention, Attitude, Behavioural intention, Convenience motivation, Perceived ease of use, Time-saving orientation, Price-saving orientation

## Abstract

**Background**: During the COVID-19 pandemic, Malaysian consumers were more likely to purchase food online and have it delivered to their doorstep. To stay afloat, many restaurants were pushed to provide online food delivery services (OFDS), and this sector has grown tremendously. However, will the trend persist after the pandemic? This study aims to look into how consumers’ perceptions of OFDS affect their attitude towards them. It investigates the relationship between convenience motivation, perceived ease of use, time-saving orientation and price-saving orientation in terms of future intent to use OFDS.

**Method**: Primary data was collected from 307 respondents in Malaysia using convenience sampling method through an online survey. Respondents’ demographic background was presented statistically in cross tabulation tables to study the ratio comparison implicitly. Consistent Partial Least Square approach and bootstrapping techniques with 5,000 subsamples was employed, with the aid of SmartPLS.V3 software, to identify the significant factors influencing consumers’ continuance intention after the pandemic.

**Result**: Perceived ease of use does not contribute significantly to continuance intention as most consumers have prior online purchase experience.  Nevertheless, time-saving orientation has a positive correlation with perceived ease of use due to the simplicity of placing an order with just a click.  It is also found that price-saving orientation is related to convenience motivation, particularly when prices can be compared on the websites or online ordering platforms. Consumers’ intention to continue using OFDS even after the COVID-19 pandemic is positively influenced by all the parameters studied, except for perceived ease of use.

**Conclusion**: Limited work has been done on the continuance intention to use OFDS beyond the pandemic. This study provides insight for food retailers on how to enhance their business and retain their customers with the support of technology, even after the COVID-19 pandemic.

## Introduction

People in the digital age rely heavily on the internet and smartphones in their daily lives. Unsurprisingly, many businesses have turned to e-commerce to stay competitive. In Malaysia, 84.2% of the population uses the internet, 88.3% of them use a shopping app each month and 6.86 million people used online food delivery services (OFDS) to order take-away food in 2020. ^1^

The lockdown implemented during the COVID-19 outbreak was enacted in order to minimise physical contact. This has forced consumers to adjust their preferences and opt for digital services, including food purchases. ^2^ As such, restaurants were eager to collaborate with online delivery platforms in order to stay in business. ^3^ GrabFood’s deliveries increased by 30%, with 8,000 new merchants whose online revenues increased by 25%. ^2^ Thus, Malaysia’s OFDS market increased tremendously in 2020, by 45.9% from 2019, and is expected to reach US$370 million in revenue over the next four years. ^1^^,^^4^

Although the OFDS industry has significant growth potential, many studies focused on consumers’ attitude towards OFDS during its initial adoption. ^5^ However, very little is known about the factors that influence consumers’ willingness to order food online regularly, particularly after a pandemic. Therefore, this study aims to further investigate the continuance intention of using OFDS beyond the COVID-19 outbreak.

## Literature review and hypotheses development

### Convenience motivation

Convenience is defined as the perceived time, value and effort required to facilitate the use of OFDS. Consumers now have the freedom to choose from a wide range of food providers listed on the internet at any time and from anywhere. As a result of its convenience, consumers will be motivated to use OFDS on a regular basis. ^6^^,^^7^

A total of 47% of e-commerce users in Southeast Asia shopped online to save time and energy, and 87% agreed on the usefulness of internet services during the COVID-19 outbreak. ^8^ Malaysians also prefer online shopping when they have a hectic schedule. ^9^ The ease of comparing prices across different online platforms and a wide variety of items are all motivating factors that drive consumers to shop online. Convenience was also cited as the top reason for shopping online in Q4 2020, and remained the top three reasons in Q1 2021. ^10^

### Perceived ease of use

Perceived ease of use (PEOU) refers to a person's perception of how hassle-free it is to use a system. The quality of a system is defined as the ease with which pages can be navigated, the presence of a clear and uncomplicated layout, and the system's dependability. ^11^ It is critical for businesses to ensure that their online platform is simple to use because bad designs or a complicated process will deter consumers from continuing with the online purchase.

The amount of effort required to use a system will serve as a critical predictor of its adoption and subsequent usefulness. ^12^^,^^13^ It was discovered that if it is relatively effortless to use a system, consumers are more likely to order food online. ^14^

### Time-saving orientation

In today's fast-paced world, where consumers’ busy schedules mean time is in short supply, time-saving orientation (TSO) has become a critical factor in easing daily tasks while fully utilising time. Many office workers could not afford the time and trouble of going out to eat, including driving and queuing up to place order. Thus, using OFDS is the quickest way to get food and the time saved can be used to complete other tasks.

Higher-income consumers value time because of the opportunity costs. As such, they find online shopping appealing because it allows them to make better use of their time. ^15^ A study discovered that timesaving is the key determinant of consumers' motivation to use technology-based self-service. ^16^ When consumers are able to save time, their perception turns positive and as a result, their attitude towards OFDS also becomes favourable. ^6^^,^^17^^,^^18^

### Price-saving orientation

Price can be defined as the value (monetary or non-monetary) an individual must put forth in an exchange for a product or service. ^19^^,^^20^ One of the key factors influencing customer satisfaction is price-saving orientation (PSO), which includes offers and discounts provided by sellers. ^21^ 82.9% of Malaysians purchased a product online in the past month. ^1^ The internet makes it easier to compare prices among different online sellers, which has proven to be advantageous for consumers to purchase at a lower price, which in turn has a significant effect on their behavioural intention to shop online. ^13^^,^^22^

OFDS provide additional perks such as not having to pay for service charge imposed by the restaurants, as well as getting free delivery and discount coupons. Additionally, consumers do not need to expend energy or effort to visit a physical store or restaurant. Thus, consumers will be more satisfied with their online food ordering experience and will be more likely to use these services in the future. ^5^^,^^18^

### Attitude, behavioural intention and continuance intention

Attitude (ATT) can be defined as a consumer's overall reaction when using a specific device or technology. ^23^ It refers to a person's reaction, whether positive or negative, to a given object. ^24^ When consumers believe that online food ordering is useful and capable of easing their daily lives, they are more likely to develop a positive attitude which will lead to continuance intention (CI) of using it. Thus, attitude is positively related to behavioural intention. ^17^^,^^25^^,^^26^

Behavioural intention (BI) is defined as a person's proclivity to act in a certain way. ^27^ The intent to use OFDS denotes a consumer's desire to purchase food and beverages through online delivery platforms. ^17^ Many studies have established that the factors used to measure BI include positive word-of-mouth, willingness to recommend a product or service to others and also repurchase intention. ^28^ Consumers who are pleased and content with their online purchase experience are expected to continue doing so. ^5^

**Figure 1.**
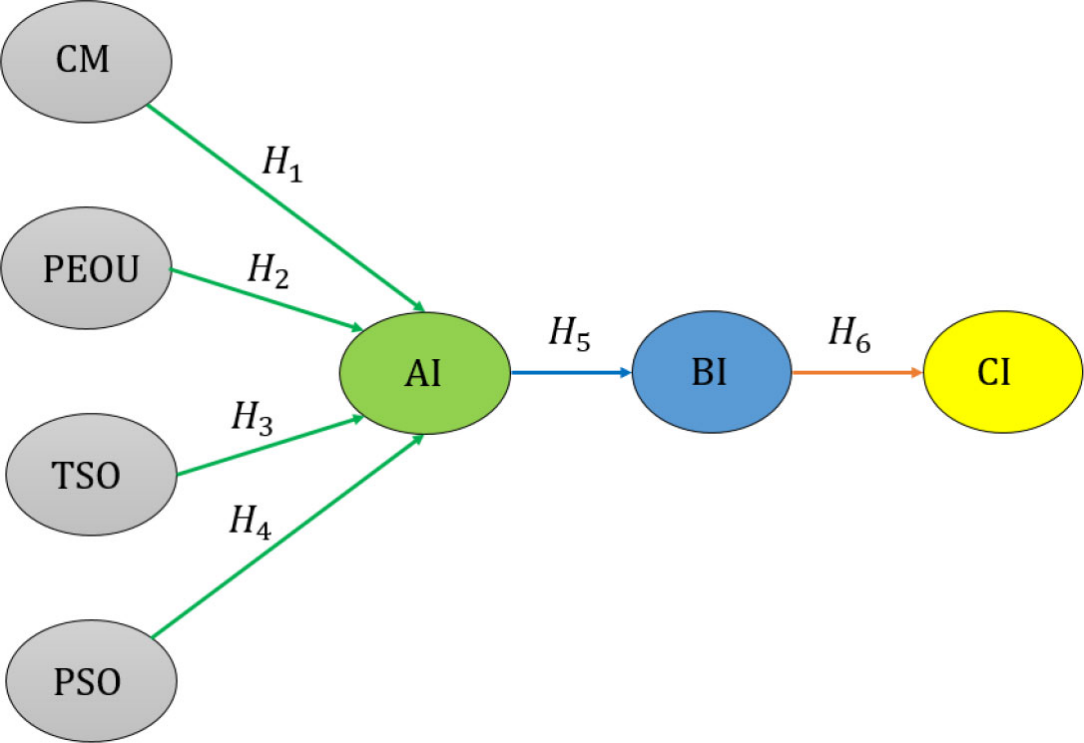
Research model.

The main objective of this study is to identify the factors that may influence consumers’ attitude and behaviour towards continuance intention in using OFDS post pandemic, as illustrated in the proposed research model in Figure 1. The hypotheses are proposed as follows:

*H1: Convenience motivation positively influences consumers’ attitude towards online food delivery services.*

*H2: Perceived ease of use positively influences consumers’ attitude towards online food delivery services.*

*H3: Time-saving orientation positively influences consumers’ attitude towards online food delivery services.*

*H4: Price-saving orientation positively influences consumers’ attitude towards online food delivery services.*

*H5: Attitude positively influences consumers’ behavioural intention towards online food delivery services.*

*H6: Behavioural intention positively influences consumers’ continuance intention towards online food delivery services.*

## Methods

### Ethics

Research ethics approval was obtained from Multimedia University, Malaysia (EA1422021) and the respondents gave their written informed consent when filling out the Google Form.

### Questionnaire development

An online survey with close-ended questions was designed using Google Form to examine the research hypotheses. It consisted of two parts: demographic information of respondents and 25 measurement items which indicated seven variables, namely, CM, PEOU, TSO, PSO, ATT, BI and CI towards OFDS, which were adopted from previous studies ^5^^,^^17^^,^^25^^,^^29–33^ and recorded in Table 1. All items were measured based on a five-point Likert-type. ^34^^,^^35^

**Table 1.** Measurement items of the study.

Constructs	Indicators		Sources
Convenience motivation	CM1:	Online food ordering would allow me to order food at any time.	Brewer and Sebby (2021) Cho *et al*. (2019) Ganesh *et al*. (2010) Troise *et al*. (2021)
	CM2:	Online food ordering would allow me to order food at any place.	
	CM3:	Online food ordering would make my daily life easier.	
	CM4:	I like the comfort of ordering food without leaving home.	
Perceived ease of use	PEOU1:	I would find that it is easy to use OFDS.	Liébana-Cabanillas *et al*. (2017) Troise *et al*. (2021)
	PEOU2:	I would find that using OFDS requires minimum effort.	
	PEOU3:	I would find that learning how to order food online is easy for me.	
	PEOU4:	I would find that it is easy to navigate through the online food ordering platform.	
Time-saving orientation	TSO1:	I believe that I can save time by using OFDS to order food.	Yeo *et al*. (2017)
	TSO2:	Using OFDS shortens the time spent to select my meal.	
	TSO3:	Using OFDS shortens the time spent to get my meal ready.	
	TSO4:	It is important for me to purchase food as quickly as possible by using OFDS.	
Price-saving orientation	PSO1:	I can save money by checking and comparing the price of different OFDS before purchase.	Yeo *et al*. (2017)
	PSO2:	Online discount coupons help me to save a lot, compared to purchasing at shop/restaurant.	
	PSO3:	I can search for cheaper food deals in different websites or online platforms.	
	PSO4:	Online food retailers offer better value for my money spent on food.	
Attitude	ATT1:	Purchasing food through OFDS is a wise action.	Yeo *et al*. (2017)
	ATT2:	Purchasing food through OFDS is a good idea.	
	ATT3:	Purchasing food through OFDS is a sensible thing to do.	
Behavioural intention	BI1:	I plan to use OFDS to order food in the future.	Cho *et al*. (2019) Troise *et al*. (2021)
	BI2:	I am willing to use OFDS to order food whenever possible.	
	BI3:	I am likely to keep using OFDS to order food.	
Continuance intention	CI1:	I intend to use OFDS continuingly after COVID-19.	Alalwan (2020) Cho et al. (2019) Zhao and Bacao (2020)
	CI2:	If I have the opportunity, I will continuingly order food through OFDS after COVID-19.	
	CI3:	I am willing to use OFDS continuingly in future.	

### Data collection

The Krejcie and Morgan sampling method was used as a guideline in estimating the sample size, and convenience sampling method was used to source participants. ^36^ This sampling method is commonly used by researchers for similar studies, such as a recent study on the intention to use OFDS among university students in Malaysia, which gathered 290 samples for data analysis. ^18^

A primary dataset of 307 respondents was gathered, in order to examine consumers’ perception and attitude towards OFDS during the pandemic, which is critical to the future growth of the OFDS industry. The respondents were close contacts (relatives, friends and students) of the authors of this study, and were invited through email, Facebook and WhatsApp, between 22 ^nd^ March 2021 and 18 ^th^ April 2021.

### Hypotheses approach

Demographic background of respondents is presented descriptively and graphically. Consistent Partial Least Square (PLSc) approach ^37–39^ was applied to study the reflective and formative factors in this study and SmartPLS.v3 software was the main tool used (a free version is available for 30 days). Reliability and validity were tested in factor analysis and bootstrapping of 5,000 subsamples was used to estimate PLSc path model.

## Results

### Profile of survey respondents

**Figure 2.**
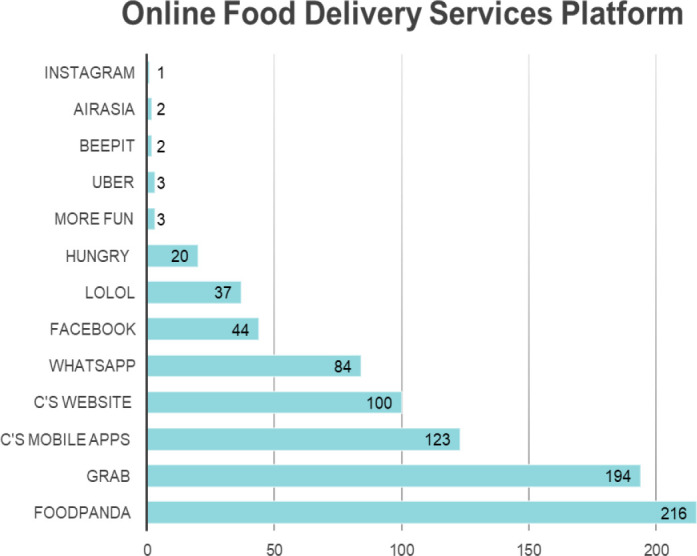
Distribution of online food delivery services platform.

Table 2 shows the demographic profile of 307 respondents. ^52^ Overall, 83.39% of respondents use OFDS and 70.68% prefer to eat at home, compared to at a restaurant. Figure 2 depicts the distribution of respondents who ordered food via third-party mobile apps, social media, or the company’s own website or mobile apps. Foodpanda (70.36%) and GrabFood (63.19%) are the most popular in Malaysia because it is user-friendly. ^40^ However, social media platforms such as Instagram are more suitable for promoting food rather than ordering. ^41^

**Table 2.** Frequency and percentage distribution of demographic profile.

Characteristics	n = 307	%
**Age**		
Under 18	4	1.30
18 ~ 25	121	39.41
26 ~ 30	47	15.31
31 ~ 40	80	26.06
41 ~ 50	40	13.03
51 ~ 60	12	3.91
60 and above	3	0.98
**State**		
Malacca	152	49.51
Johor	71	23.13
Selangor	41	13.36
Negeri Sembilan	14	4.56
Kelantan	7	2.28
Pahang	5	1.63
Perak	5	1.63
Sarawak	5	1.63
Penang	4	1.30
Kedah	3	0.98
**Use OFDS**		
Yes	256	83.39
No	51	16.61
**Dining preference**		
Outside	90	29.32
At home	217	70.68

Table 3 recorded the feedback of the respondents whereby the mode for all measurement items is “Agree”, which contributes to the left-skewed distribution except PSO4. The average and standard deviation of variables are recorded in Table 4 and each average is close to “4” (Agree) except PSO.

**Table 3.** Feedback of the respondents.

	Strongly disagree	Disagree	Neutral	Agree	Strongly agree
CM1	5	11	59	151	81
CM2	6	29	64	134	74
CM3	6	7	50	159	85
CM4	5	10	63	140	89
PEOU1	5	6	62	179	55
PEOU2	5	8	77	164	53
PEOU3	5	7	52	167	76
PEOU4	4	14	71	160	58
TSO1	8	11	62	151	75
TSO2	11	30	80	137	49
TSO3	7	17	81	151	51
TSO4	6	20	81	137	63
PSO1	20	45	89	108	45
PSO2	13	29	86	119	60
PSO3	10	26	91	135	45
PSO4	19	51	106*	91	40
AI1	3	9	101	149	45
AI2	3	9	77	170	48
AI3	5	10	101	158	33
BI1	5	6	96	154	46
BI2	4	14	84	162	43
BI3	7	14	99	142	45
CI1	4	22	93	139	49
CI2	3	22	82	155	45
CI3	5	16	87	149	50

**Table 4.** Mean and standard deviation of the variables.

	Mean	SD
CM	3.93	0.73
PEOU	3.88	0.71
TSO	3.74	0.79
PSO	3.45	0.93
AI	3.74	0.72
BI	3.72	0.76
CI	3.70	0.82

Table 5 shows the ratio comparison of using OFDS and dining preference based on age, gender, marital status and personal income level. OFDS usage among single young adults (>85% each age group below 40 years old; single 86%) is higher compared to married adults (77%) during pandemic than before the pandemic. ^11^ Elderly or married adults prefer to enjoy their food at home. Although 71.34% of the respondents were earning a low income, they still preferred to use OFDS (84%) and dine at home (71%) compared to higher income respondents. The statistics revealed a significant difference in online food ordering trends between age groups, but not between genders.

**Table 5.** Comparison of OFDS usage and dining preference.

Characteristic	Ratio	
	OFDS usage	Dining at home
**Age**		
< 18	1.00	0.50
18 ~ 25	0.86	0.67
26 ~ 30	0.85	0.57
31 ~ 40	0.88	0.71
41 ~ 50	0.65	0.85
51 ~ 60	0.83	1.00
> 60	0.33	1.00
**Gender**		
Female	0.83	0.74
Male	0.85	0.63
**Marital status**		
Single	0.86	0.67
Married	0.77	0.77
Others	1.00	1.00
**Personal income level**		
B40	0.84	0.71
M40	0.83	0.67
T20	0.70	0.70

## Measurement of model

### Reliability and validity

Table 6 shows Cronbach’s alpha ^42^^,^^43^ and composite reliability (CR) ^37^^,^^44^^,^^45^ for each variable as above 0.8, which indicates good internal consistency of the questionnaire’s questions scale in measuring a similar variable. * indicates CR>0.95 but there are no significant changes after its removal. ^37^ The average variance extracted (AVE) indices ^46^ are greater than 0.6 for each variable, indicating no convergent validity problems.

**Table 6.** Cronbach’s alpha, composite reliability and average variance extracted.

	Cronbach’s alpha	Composite reliability	AVE	Item
CM	0.831	0.831	0.553	4
PEOU	0.909	0.909	0.713	4
TSO	0.877	0.877	0.642	4
PSO	0.915	0.915	0.729	4
ATT	0.924	0.925	0.804	3
BI	0.910	0.910	0.771	3
CI	0.959	**0.959** *	0.887	3

* indicates CR > 0.95.

In Table 7 Fornell-Larcker criterion, ^46^^,^^47^ the diagonals represent the square root of AVE and off diagonals represent the coefficient of correlation. One tail t-test is conducted on the coefficient of correlation at 5% level of significance. The results revealed that there is a positive correlation between the variables with *p*-value of 0. There are no discriminant validity issues with the support of HTMT values, recorded in Table 8 based on HTMT _0.85_ criterions.

**Table 7.** Fornell-Larcker criterion.

	CM	PEOU	TSO	PSO	ATT	BI	CI
**CM**	0.74						
**PEOU**	0.77	0.84					
**TSO**	0.74	0.70	0.80				
**PSO**	0.53	0.54	0.62	0.85			
**ATT**	0.66	0.58	0.66	0.56	0.90		
**BI**	0.73	0.62	0.65	0.55	0.82	0.88	
**CI**	0.58	0.57	0.64	0.54	0.75	0.83	0.94

**Table 8.** Heterotrait-Monotrait ratio (HTMT).

	CM	PEOU	TSO	PSO	ATT	BI	CI
**CM**							
**PEOU**	0.77						
**TSO**	0.74	0.70					
**PSO**	0.53	0.54	0.62				
**ATT**	0.66	0.58	0.66	0.56			
**BI**	0.73	0.62	0.65	0.54	0.82		
**CI**	0.58	0.57	0.64	0.54	0.75	0.83	

### Consistent partial least square (PLSc) path modelling approach

Six hypotheses were tested using PLSc, ^39^ a variance-based structural equation modelling technique, with no concerns about distribution or multicollinearity. In the past decade, the use of PLS modelling has gradually increased in order to handle more complex models.

**Figure 3.**
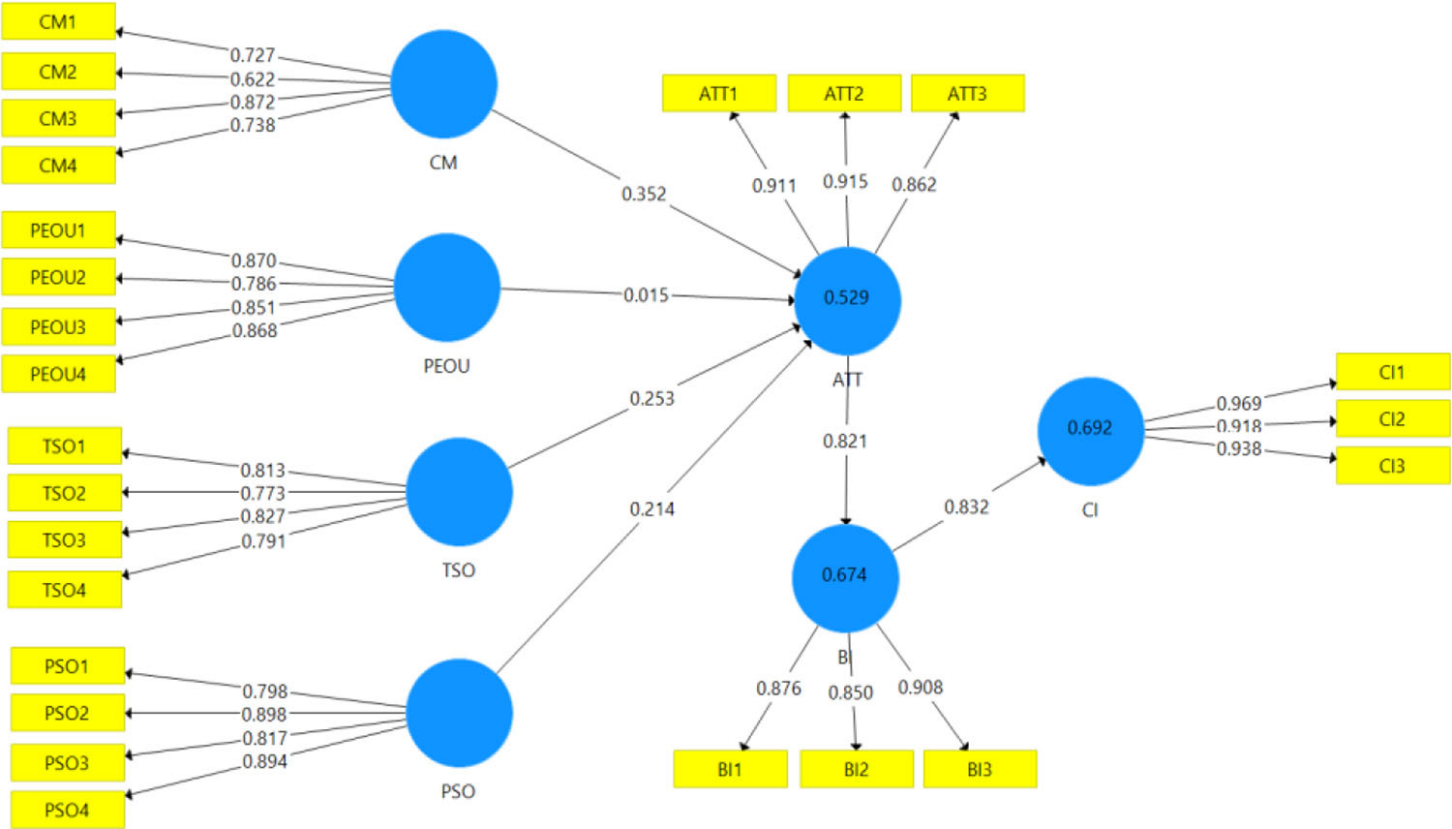
Part coefficient and outer loading.

Table 9 summarises the result of the hypotheses presented in Figure 3, which indicates the path coefficient and outer loading of the variable. PEOU is found to be insignificant in influencing consumers’ attitude towards OFDS ( *p*-value > 0.05). Consumers’ attitude towards using OFDS during and post the COVID-19 pandemic is, however, positively influenced by CM ( *p*-value < 0.05), TSO ( *p*-value < 0.05) and PSO ( *p*-value < 0.05). Furthermore, hypotheses of ATT positively influencing consumers’ BI ( *p*-value < 0.05) and also BI positively influencing consumers’ CI ( *p*-value < 0.05) towards OFDS are supported in this study. Thus, H1, H3, H4, H5 and H6 are validated while H2 is rejected.

**Table 9.** Summary of hypotheses testing.

Hypothesis	Path	t-value	*p*-value	Decisions
H1	CM-->ATT	2.648	0.008	Supported
H2	PEOU-->ATT	0.116	0.907	Rejected
H3	TSO-->ATT	2.326	0.020	Supported
H4	PSO-->ATT	2.773	0.006	Supported
H5	ATT-->BI	24.220	0.000	Supported
H6	BI-->AI	27.284	0.000	Supported

## Discussion

Based on the findings of this study, convenience motivation has a significant impact on consumers’ attitude towards OFDS, which is consistent with previous studies. ^6^^,^^8^^-^^10^^,^^17^^,^^21^^,^^25^^,^^29^ OFDS platforms are very well developed nowadays, enabling consumers to order food online at any time and from any location, without having to leave home. With just a click and via a cashless payment system, food will be ready in a short period of time, providing consumers with a great deal of convenience. However, electronic devices have already been integrated into our daily routines for a long time and people are already familiar with these devices, thus perceived ease of use is not a significant motivator that would influence consumers to continue ordering food online. ^5^^,^^6^^,^^13^^,^^48^

Time is an important factor that consumers, particularly working adults and students, are concerned about. ^6^^,^^17^^,^^18^ Consumers are eager to use OFDS because they can save a significant amount of time from menu selection to food preparation. Especially during rush hour, OFDS will be their first choice rather than waiting in line at a restaurant. OFDS also saves consumers money, as they can compare the prices offered by different food retailers and budget for a meal. Food retailers must continue to offer competitive price, such as giving attractive discount coupons or free delivery services to influence consumers to revisit. ^21^ With the assistance of third-party apps, price-saving orientation significantly influences consumers’ attitude towards OFDS continuance intention after the pandemic, ^17^ but perhaps not for all students. ^18^

Previous studies conducted in this field of study have focused on the general intention of using OFDS. ^25^^,^^33^^,^^48^ This paper, however, investigates consumers’ attitude and behaviour regarding their continuance intention of using OFDS after the COVID-19 pandemic. The left-skewed distribution of continuance intention’s measurement items significantly indicates that there is a high possibility of consumers using OFDS continuously after COVID-19, and this supports the hypothesis that a positive behavioural intention will lead to continuance of using a service. A satisfying online shopping experience fosters a positive attitude toward using the services and, as a result, always increases the likelihood of future purchase behaviour. ^49–51^

### Limitations

This study did not take into account all of the possible factors that might influence the continuance intention of using OFDS after the pandemic. The model could be improved in the future by including more variables, such as, customer satisfaction and social influences. Furthermore, the findings cannot be generalised as a whole due to convenience sampling biasness. In the future, the study could be narrowed down to a specific group; perhaps looking at some larger cities with higher demand and supply for OFDS.

## Conclusions

OFDS is a consumer-focused market which aims to bring comfort to consumers so that they are able to get their favourite food at the best price and convenience without having to leave home. This is consistent with our findings that convenience motivation, time-saving orientation and price-saving orientation were the primary factors influencing consumers’ attitude towards OFDS during and post the COVID-19 pandemic. The findings also revealed that consumers who have a positive attitude and behaviour towards OFDS tend to have favourable feedback on the continuance intention after COVID-19.

Nevertheless, although results showed that there is a significant impact on the continuance intention towards OFDS after COVID-19, there are several issues and challenges that need to be addressed. Food retailers should consider how to retain the food quality and ensure fast delivery when orders increase. They should also look into collaboration with third-party apps such as GrabFood and Foodpanda to help boost their sales and maximise profits. We believe that consumers will soon adopt OFDS into their lifestyle, making it a norm, after the pandemic. Therefore, it is crucial for food retailers to work in this direction to sustain and grow their business model.

## Data availability

### Underlying data

Figshare: Online Food Delivery Service.

DOI: http://doi.org/10.6084/m9.figshare.14772951. ^52^

This project contains the following underlying data:

• Data file 1. (Survey results, CVS format)

### Extended data

Figshare: Online Food Delivery Service Questionnaire 2021

DOI: http://doi.org/10.6084/m9.figshare.16566414. ^53^

This project contains the following extended data:

• Data file 1. (Survey questions, CVS format)

Data are available under the terms of the Creative Commons Zero “No rights reserved” data waiver (CC0 1.0 Public domain dedication).
