# Online food delivery services: cross-sectional study of consumers’ attitude in Malaysia during and after the COVID-19 pandemic

**DOI:** 10.12688/f1000research.73014.3

**Published:** 2026-03-03

**Authors:** Sin Yin Tan, Su Yin Lim, Sook Fern Yeo

**Affiliations:** 1 Faculty of Information Science and Technology, Multimedia University, Melaka, 75450, Malaysia; 2 Faculty of Business, Multimedia University, Melaka, 75450, Malaysia

**Keywords:** Online food delivery services, Continuance intention, Attitude, Behavioural intention, Convenience motivation, Perceived ease of use, Time-saving orientation, Price-saving orientation

## Abstract

**Background**: During the COVID-19 pandemic, Malaysian consumers were more likely to purchase food online and have it delivered to their doorstep. To stay afloat, many restaurants were pushed to provide online food delivery services (OFDS), and this sector has grown tremendously. However, will the trend persist after the pandemic? This study aims to look into how consumers’ perceptions of OFDS affect their attitude towards them. It investigates the relationship between convenience motivation, perceived ease of use, time-saving orientation and price-saving orientation in terms of future intent to use OFDS.

**Method**: Primary data was collected from 307 respondents in Malaysia using purposive sampling method through an online survey. Respondents’ demographic background was presented statistically in cross tabulation tables to study the ratio comparison implicitly. Consistent Partial Least Square approach and bootstrapping techniques with 5,000 subsamples was employed, with the aid of SmartPLS.V3 software, to identify the significant factors influencing consumers’ continuance intention after the pandemic.

**Result**: Perceived ease of use does not contribute significantly to continuance intention as most consumers have prior online purchase experience.  Nevertheless, time-saving orientation has a positive correlation with perceived ease of use due to the simplicity of placing an order with just a click.  It is also found that price-saving orientation is related to convenience motivation, particularly when prices can be compared on the websites or online ordering platforms. Consumers’ intention to continue using OFDS even after the COVID-19 pandemic is positively influenced by all the parameters studied, except for perceived ease of use.

**Conclusion**: Limited work has been done on the continuance intention to use OFDS beyond the pandemic. This study provides insight for food retailers on how to enhance their business and retain their customers with the support of technology, even after the COVID-19 pandemic.

## Introduction

The COVID-19 pandemic, which swept the world in 2020, caused an unprecedented gripping death toll, affecting the public health, food systems and workplace. Tens of millions of people face the looming threat of extreme poverty, while millions of businesses are on the brink of closure. Nearly half of the world’s workplace, totaling 3.3 billion people, are at risk of unemployment. ^1^ In Malaysia, the pandemic had a profound impact on the nation’s economy, labour market, and social dynamics. The unemployment rate rose from 1.2% to 4.5% in 2020, the highest in nearly three decades. Many people have lost their jobs, sources of income, and even businesses as a result of this situation. ^2^

This state of affairs is extremely concerning and may jeopardize the achievement of the sustainable development goals (SDGs) established by the United Nations in 2015. In particular, SDG 1 targets the eradication of extreme poverty in all forms everywhere by 2030. Among others, the outcome goals are to lift individuals living on less than US$1.90 per day out of poverty and to reduce all poverty by half. Even though global poverty has been steadily declining for the last 20 years, research by the UNU World Institute for Development Economics Research cautioned that the COVID-19 pandemic might raise it to 8% of the world’s population in just a few months into the pandemic. ^3^

Everyone must do their part to overcome the challenges of COVID-19, including the government, the commercial sector, and the general public. If businesses, especially, could modify their business models to cater to the population at the bottom of the pyramid, they could play a significant role in alleviating poverty while still profiting. Businesses may reach spectacular new markets made up of billions of people at the lower end of the income spectrum thanks to the web and e-commerce, which are made possible by the widespread use of mobile devices to access the internet in this digital age. ^4^ Unsurprisingly, many businesses have turned to e-commerce to stay competitive. In Malaysia, 84.2% of the population uses the internet, 88.3% of them use a shopping app each month and in particular, 6.86 million people used online food delivery services (OFDS) to order take-away food in 2020. ^5^

The COVID-19 outbreak lockdown, enacted to minimise physical contact, has forced consumers to adjust their preferences, increasingly turning to digital services for various needs, including food purchases. ^6^ As such, restaurants were eager to collaborate with online delivery platforms in order to stay in business. ^7^ This avenue not only ensures their continuity but also provides a platform for small and medium-sized enterprises (SMEs) seeking to extend their reach in the online sphere. GrabFood’s deliveries increased vastly by 30%, with 8,000 new merchants whose online revenues increased by 25%. ^6^ Malaysia’s OFDS market undoubtedly, increased tremendously in 2020, by 45.9% from 2019, and is expected to reach US$370 million in revenue over the next four years. ^5^ ^,^ ^8^ Apart from preventing business closures, e-commerce also plays an important role in creating job opportunities, especially for those who have lost their source of income as a result of the pandemic. While approximately 25% of GrabFood’s deliveries were made by GrabCar drivers, who were hampered by the limited movement, Foodpanda reported a 7.5% rise in new riders during the lockdown. Over 10,000 people joined Grab as drivers and delivery partners, in reality, opening up employment chances for those in need. ^6^

The OFDS industry has demonstrated remarkable growth potential, ^9^ a trend notably accentuated by the events of 2020. Therefore, it is imperative to explore the determinants influencing consumers’ inclination to order food online on a regular basis, particularly in the aftermath of a pandemic. Amid the global pandemic, the lockdown, which leaves consumers with no choice but to prepare their own meals or order them online has resulted in an unprecedented surge in the OFDS business in 2020. A pertinent question arises as to whether this surge was a temporary phenomenon or if it will lead to a sustained growth in the long term. Research must explore the factors influencing consumers’ willingness to embrace online food ordering as a routine practice, even as pandemic restrictions subside. This would assist food retailers in positioning their products and services to capitalise on this emerging market.

Previous research has primarily focused on consumers’ attitude towards online services in general, with only a few researchers focusing on consumer experiences with OFDS. ^10^ ^,^ ^11^ Despite the fact that online food delivery is an emerging trend, the majority of the studies in this domain examined consumers’ intention and initial adoption of OFDS. ^9^ ^,^ ^12^ ^,^ ^13^ Some researchers investigated factors such as customer satisfaction, ^11^ ^,^ ^12^ ^,^ ^14^ ^–^ ^16^ convenience, ^10^ ^,^ ^11^ ^,^ ^13^ ^,^ ^17^ ^,^ ^18^ perceived ease of use, ^13^ ^,^ ^17^ ^,^ ^18^ price-saving, ^10^ ^,^ ^11^ ^,^ ^17^ ^,^ ^19^ ^–^ ^21^ customer experience, ^11^ ^,^ ^13^ product information quality, ^17^ ^,^ ^21^ ^,^ ^22^ prior online purchase experience, ^10^ ^,^ ^20^ perceived usefulness ^18^ ^,^ ^20^ and perceived trust ^14^ ^,^ ^18^ in using OFDS. However, very little research has been conducted to investigate the continuance intention of OFDS in this unprecedented pandemic state that may intensify usage. ^14^ ^,^ ^16^ Will consumers continue to order food online once the restrictions on movement are lifted? Therefore, to bridge this gap, this study aims to further investigate the critical factors that consumers believe are important in motivating them to continue using OFDS after the COVID-19 epidemic.

## Literature review and hypotheses development

### Theoretical background

This study aims to examine the essential factors perceived by consumers as influential in their decision to persist in using OFDS following the COVID-19 outbreak. Previous studies have frequently combined the Theory of Planned Behaviour (TPB) and Technology Acceptance Model (TAM) to explain why people engage in a specific behaviour. For example, a study conducted in China used TPB, TAM and three patient-centered factors to examine the elements affecting patients’ acceptance of mobile medical platforms, ^23^ while another study conducted in Italy combined TPB and TAM to analyse the main drivers of users’ intention to use foods delivery apps. ^18^ Other examples include the examination of continuance intention to utilise mobile banking in Jordan, achieved through the integration of UTAUT, TPB, TAM and service quality with machine learning methods, ^24^ and understanding library user behavioural utilisation intention of physical book as compared to e-book format in Malaysia by combining TAM, TPB and Theory of Self-Regulation (TSR). ^25^

This study proposes a similar approach to form an integrative theoretical research model adapted from TAM and TPB, expanding it to include three additional constructs: convenience motivation (CM), time-saving orientation (TSO), and price-saving orientation (PSO). TAM, conceptualised by Davis in 1989, put forward that users’ attitudes toward a technology are shaped by their perception of its ease of use and usefulness. These attitudes subsequently influence users’ behavioural intentions to adopt and continue using the technology. ^26^ In the context of OFDS, perceived ease of use relates to how easy consumers believe ordering food online is. A user-friendly interface, straightforward navigation, and intuitive app design all contribute to a high level of perceived ease of use. Consumers who find the service easy to use are more likely to form positive attitudes and intentions toward its use.

TPB, developed by Ajzen in 1991 extends the understanding of user behaviour by incorporating attitudes, subjective norms and perceived behavioural control as determinants of behavioural intentions, which in turn impact actual behaviour. ^27^ In the context of OFDS, consumers’ attitudes are primarily shaped by two key factors: their perceptions of the benefits, which encompass aspects such as convenience, cost-effectiveness, and time efficiency; and their perceptions of the ease of using the service. ^28^ When consumers hold favourable beliefs regarding these factors, it leads to the development of positive attitudes, which in turn, significantly enhances the likelihood of consumers forming a positive intention to engage in OFDS. In essence, attitude acts as a critical determinant in the decision-making process regarding the adoption and utilisation of OFDS among consumers.

This study examines the continued use of OFDS among experienced users, a context where the typical drivers of the TPB may differ from those relevant during initial adoption. At this post-adoption stage, Perceived Behavioural Control (PBC) is less critical because users already have the skills, resources, and access needed to use the platform. Likewise, Subjective Norms (SN) play a limited role, as food ordering is usually a private, routine activity. Prior research suggests that once a behaviour becomes habitual and voluntary, consumers’ attitudes and practical evaluations tend to outweigh external pressures or perceived control. ^29– 32^ Accordingly, the model retains the attitudinal and behavioural intention components of TPB while extending it to include factors that capture the real, practical value users derive from OFDS in their daily lives.

To reflect post-pandemic priorities, the model incorporates three additional constructs: convenience motivation, time-saving orientation, and price-saving orientation. These variables capture users’ desire to simplify daily routines, save time, and gain economic benefits. Including them allows the framework to account for the “post-pandemic shift,” showing how OFDS use has evolved from a temporary, pandemic-driven behaviour into a habitual, integrated part of everyday life.

Overall, the framework integrates TAM and TPB with these three additional variables, offering a comprehensive lens on consumer behaviour. This combination considers both the technological adoption aspects and the socio-psychological factors that influence attitudes and intentions, providing insight into how usability, convenience, efficiency, and economic considerations interact to shape continued engagement with OFDS.

### Convenience motivation

Convenience is defined as the perceived time, value and effort required to facilitate the use of OFDS. Consumers now have the freedom to choose from a wide range of food providers listed on the internet at any time and from anywhere. As a result of its convenience, consumers will be motivated to use OFDS on a regular basis. ^33, 34^

A total of 47% of e-commerce users in Southeast Asia shopped online to save time and energy, and 87% agreed on the usefulness of internet services during the COVID-19 outbreak. ^35^ Malaysians also prefer online shopping when they have a hectic schedule. ^36^ The ease of comparing prices across different online platforms and a wide variety of items are all motivating factors that drive consumers to shop online. Convenience was also cited as the top reason for shopping online in Q4 2020, and remained the top three reasons in Q1 2021. ^37^

### Perceived ease of use

Perceived ease of use (PEOU) refers to a person’s perception of how hassle-free it is to use a system. The quality of a system is defined as the ease with which pages can be navigated, the presence of a clear and uncomplicated layout, and the system’s dependability. ^38^ It is critical for businesses to ensure that their online platform is simple to use because bad designs or a complicated process will deter consumers from continuing with the online purchase.

The amount of effort required to use a system will serve as a critical predictor of its adoption and subsequent usefulness. ^17^^,^^26^ It was discovered that if it is relatively effortless to use a system, consumers are more likely to order food online. ^13^

### Time-saving orientation

In today’s fast-paced world, where consumers’ busy schedules mean time is in short supply, time-saving orientation (TSO) has become a critical factor in easing daily tasks while fully utilising time. Many office workers could not afford the time and trouble of going out to eat, including driving and queuing up to place order. Thus, using OFDS is the quickest way to get food and the time saved can be used to complete other tasks.

Higher-income consumers value time because of the opportunity costs. As such, they find online shopping appealing because it allows them to make better use of their time. ^19^ A study discovered that timesaving is the key determinant of consumers’ motivation to use technology-based self-service. ^39^ When consumers are able to save time, their perception turns positive and as a result, their attitude towards OFDS also becomes favourable. ^10^^,^^20^^,^^33^

### Price-saving orientation

Price can be defined as the value (monetary or non-monetary) an individual must put forth in an exchange for a product or service. ^40^^,^^41^ One of the key factors influencing customer satisfaction is price-saving orientation (PSO), which includes offers and discounts provided by sellers. ^11^ 82.9% of Malaysians purchased a product online in the past month. ^5^ The internet makes it easier to compare prices among different online sellers, which has proven to be advantageous for consumers to purchase at a lower price, which in turn has a significant effect on their behavioural intention to shop online. ^17^^,^^42^

OFDS provide additional perks such as not having to pay for service charge imposed by the restaurants, as well as getting free delivery and discount coupons. Additionally, consumers do not need to expend energy or effort to visit a physical store or restaurant. Thus, consumers will be more satisfied with their online food ordering experience and will be more likely to use these services in the future. ^12^^,^^20^

### Attitude, behavioural intention and continuance intention

Attitude (ATT) can be defined as a consumer’s overall reaction when using a specific device or technology. ^27^ It refers to a person’s reaction, whether positive or negative, to a given object. ^43^ When consumers believe that online food ordering is useful and capable of easing their daily lives, they are more likely to develop a positive attitude which will lead to continuance intention (CI) of using it. Thus, attitude is positively related to behavioural intention. ^10^^,^^18^^,^^44^

Behavioural intention (BI) is defined as a person’s proclivity to act in a certain way. ^45^ The intent to use OFDS denotes a consumer’s desire to purchase food and beverages through online delivery platforms. ^10^ Many studies have established that the factors used to measure BI include positive word-of-mouth, willingness to recommend a product or service to others and also repurchase intention. ^46^ Consumers who are pleased and content with their online purchase experience are expected to continue doing so. ^12^

**Figure 1.**
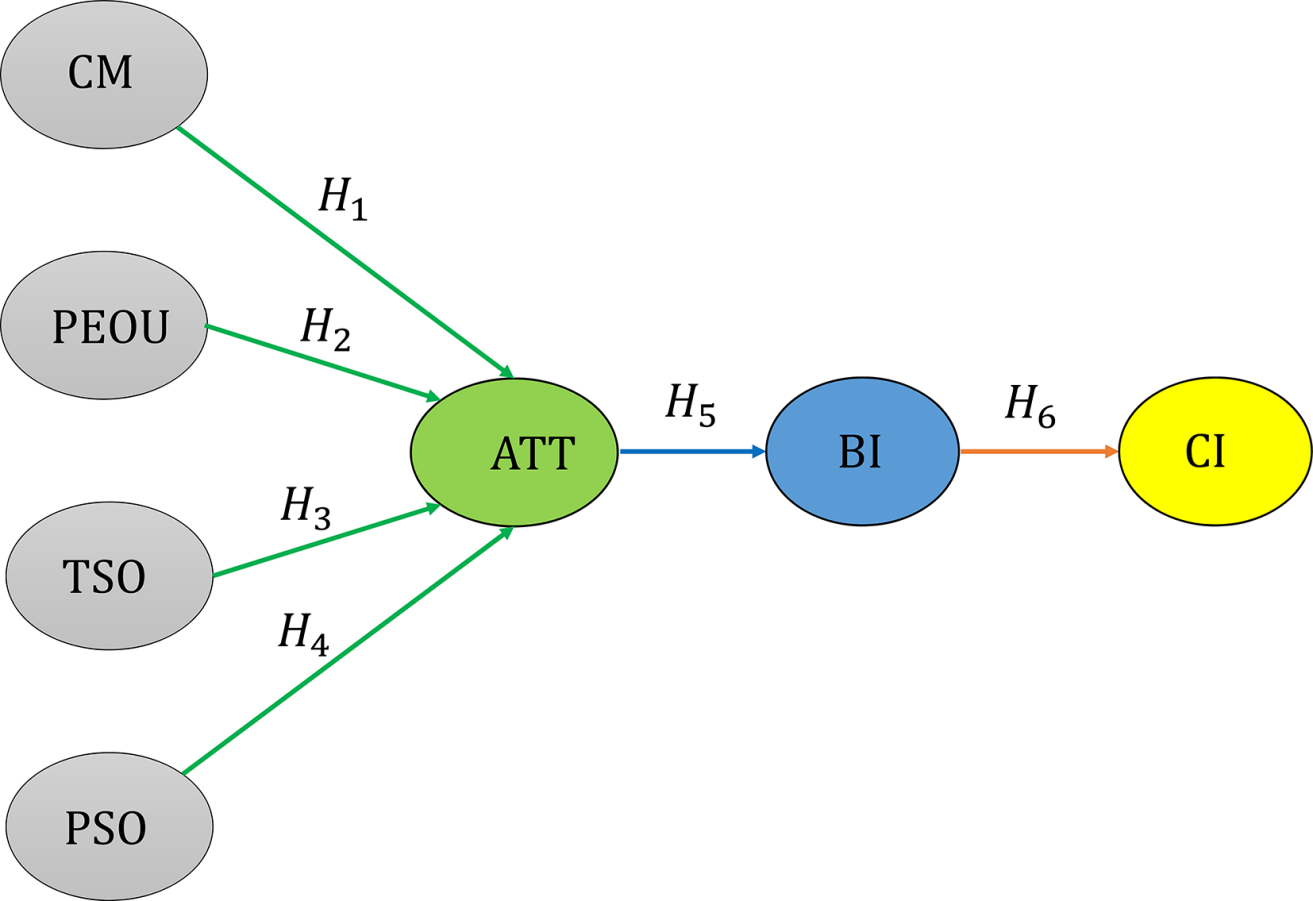
Research model.

The main objective of this study is to identify the factors that may influence consumers’ attitude and behaviour towards continuance intention in using OFDS post pandemic, as illustrated in the proposed research model in Figure 1. The hypotheses are proposed as follows:

*H1: Convenience motivation positively influences consumers’ attitude towards online food delivery services.*

*H2: Perceived ease of use positively influences consumers’ attitude towards online food delivery services.*

*H3: Time-saving orientation positively influences consumers’ attitude towards online food delivery services.*

*H4: Price-saving orientation positively influences consumers’ attitude towards online food delivery services.*

*H5: Attitude positively influences consumers’ behavioural intention towards online food delivery services.*

*H6: Behavioural intention positively influences consumers’ continuance intention towards online food delivery services.*

## Methods

### Ethics

Research ethics approval was obtained from Multimedia University, Malaysia (EA1422021) and the respondents gave their written informed consent when filling out the Google Form.

### Questionnaire development

An online survey with close-ended questions was designed using Google Form to examine the research hypotheses. It consisted of two parts: demographic information of respondents and 25 measurement items which indicated seven variables, namely, CM, PEOU, TSO, PSO, ATT, BI and CI towards OFDS, which were adopted from previous studies ^10^^,^^12^^,^^14^^,^^18^^,^^22^^,^^47^^–^^49^ and recorded in Table 1. All items were measured based on a five-point Likert-type ^50^^,^^51^ ranging from 1 (“Strongly Disagree”) to 5 (“Strongly Agree”).

**Table 1.** Measurement items of the study.

Constructs	Indicators		Sources
Convenience motivation	CM1:	Online food ordering would allow me to order food at any time.	Brewer and Sebby (2021) Cho *et al*. (2019) Ganesh *et al*. (2010) Troise *et al*. (2021)
	CM2:	Online food ordering would allow me to order food at any place.	
	CM3:	Online food ordering would make my daily life easier.	
	CM4:	I like the comfort of ordering food without leaving home.	
Perceived ease of use	PEOU1:	I would find that it is easy to use OFDS.	Liébana-Cabanillas *et al*. (2017) Troise *et al*. (2021)
	PEOU2:	I would find that using OFDS requires minimum effort.	
	PEOU3:	I would find that learning how to order food online is easy for me.	
	PEOU4:	I would find that it is easy to navigate through the online food ordering platform.	
Time-saving orientation	TSO1:	I believe that I can save time by using OFDS to order food.	Yeo *et al*. (2017)
	TSO2:	Using OFDS shortens the time spent to select my meal.	
	TSO3:	Using OFDS shortens the time spent to get my meal ready.	
	TSO4:	It is important for me to purchase food as quickly as possible by using OFDS.	
Price-saving orientation	PSO1:	I can save money by checking and comparing the price of different OFDS before purchase.	Yeo *et al*. (2017)
	PSO2:	Online discount coupons help me to save a lot, compared to purchasing at shop/restaurant.	
	PSO3:	I can search for cheaper food deals in different websites or online platforms.	
	PSO4:	Online food retailers offer better value for my money spent on food.	
Attitude	ATT1:	Purchasing food through OFDS is a wise action.	Yeo *et al*. (2017)
	ATT2:	Purchasing food through OFDS is a good idea.	
	ATT3:	Purchasing food through OFDS is a sensible thing to do.	
Behavioural intention	BI1:	I plan to use OFDS to order food in the future.	Cho *et al*. (2019) Troise *et al*. (2021)
	BI2:	I am willing to use OFDS to order food whenever possible.	
	BI3:	I am likely to keep using OFDS to order food.	
Continuance intention	CI1:	I intend to use OFDS continuingly after COVID-19.	Alalwan (2020) Cho et al. (2019) Zhao and Bacao (2020)
	CI2:	If I have the opportunity, I will continuingly order food through OFDS after COVID-19.	
	CI3:	I am willing to use OFDS continuingly in future.	

### Data collection

In this study, purposive sampling method was applied ^52^^–^^54^ because the selected samples are more representative of the population. It is commonly used by researchers for similar studies, such as a recent study on the intention to use OFDS among consumers in Malaysia, which gathered 224 samples for data analysis. ^10^ Questionnaire was sent to potential respondents who were close contacts (relatives, friends and students) of the authors of this study, and they were invited through email, Facebook and WhatsApp, between 22 March 2021 and 18 April 2021. A screening question was included to ensure that only participants with experience using OFDS were able to complete the survey.

A primary dataset of 256 respondents was gathered, in order to examine consumers’ perception and attitude towards OFDS during the pandemic, which is critical to the future growth of the OFDS industry. The minimum sample size of 191 is determined according to the guideline of Hair *et al.*, ^55^ with a maximum of 4 arrows pointing at a latent variable and minimum R ^2^ of 0.10.

### Hypotheses approach

Demographic background of respondents is presented descriptively and graphically. Consistent Partial Least Square (PLSc) approach ^55– 57^ was applied to study the reflective and formative factors in this study and SmartPLS.v3 software was the main tool used (a free version is available for 30 days). Reliability and validity were tested in factor analysis and bootstrapping of 5,000 subsamples was used to estimate PLSc path model. ^58^

## Results

### Profile of survey respondents

**Figure 2.**
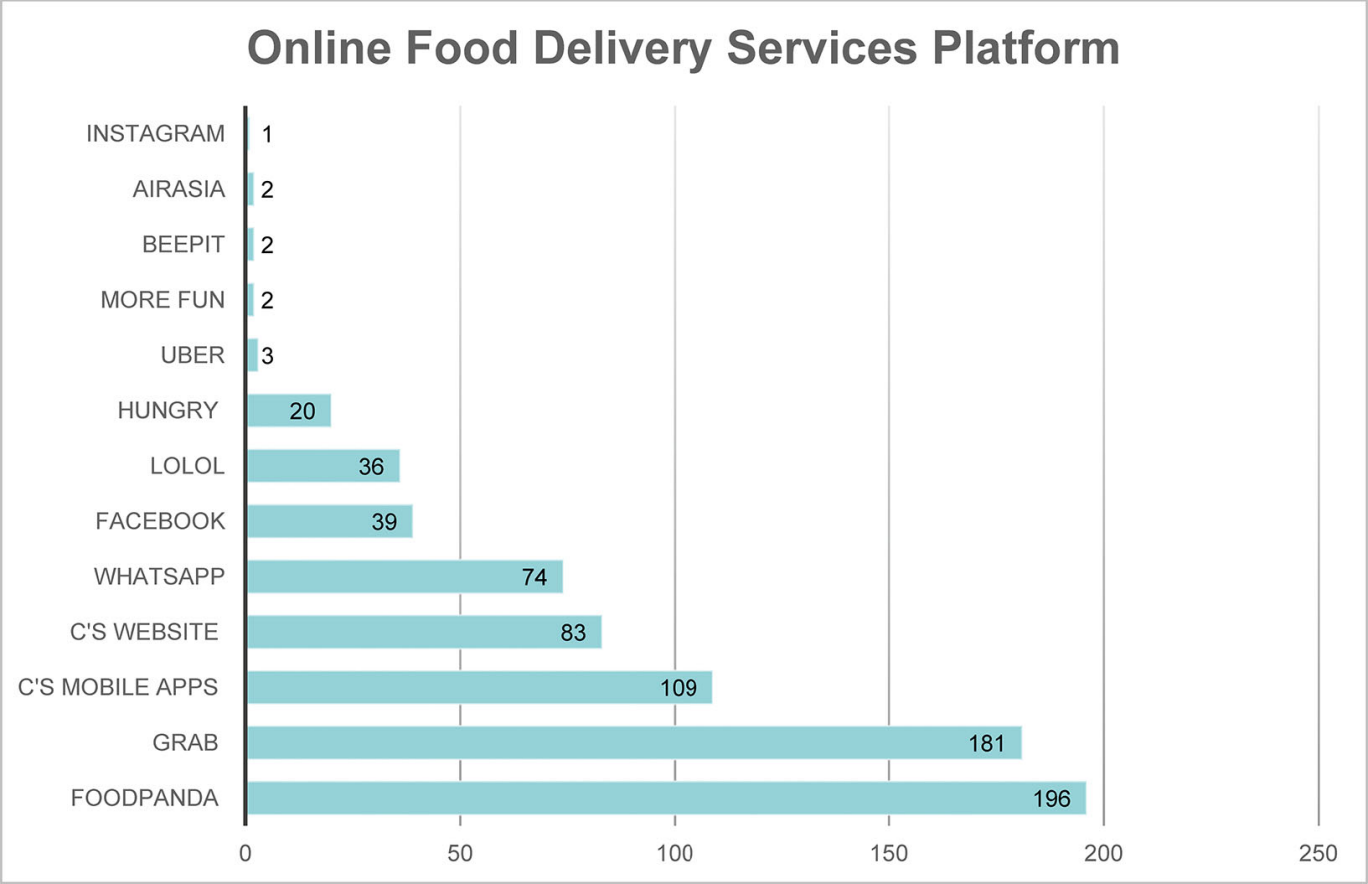
Distribution of online food delivery services platform.

Table 2 shows the demographic profile of 256 respondents. ^59^ All of them has experienced using OFDS and mostly are young adults between the age of 18 to 25 years old (40.63%). 68.75% preferred to eat at home, compared to at a restaurant. Figure 2 depicts the distribution of respondents who ordered food via third-party mobile apps, social media, or the company’s own website or mobile apps. Foodpanda (76.56%) and GrabFood (70.70%) are the most popular in Malaysia because consumers deemed that the platforms are user-friendly. ^15^ However, social media platforms such as Instagram are more suitable for promoting food rather than ordering. ^60^

**Table 2.** Frequency and percentage distribution of demographic profile.

Characteristics	n = 256	%
**Age**		
Under 18	4	1.56
18 ~ 25	104	40.63
26 ~ 30	40	15.63
31 ~ 40	71	27.73
41 ~ 50	26	10.16
51 ~ 60	10	3.90
60 and above	1	0.39
**State**		
Malacca	122	47.66
Johor	65	25.39
Selangor	38	14.85
Negeri Sembilan	10	3.91
Kelantan	5	1.95
Perak	5	1.95
Sarawak	4	1.56
Kedah	3	1.17
Pahang	2	0.78
Penang	2	0.78
**Dining preference**		
Outside	80	31.25
At home	176	68.75

Table 3 recorded the feedback of the respondents whereby the mode for all measurement items is “Agree”, which contributes to the left-skewed distribution except PSO4. The average and standard deviation of variables are recorded in Table 4 and each average is close to “4” (Agree) except PSO.

**Table 3.** Feedback of the respondents.

	Strongly disagree	Disagree	Neutral	Agree	Strongly agree
CM1	5	9	46	126	70
CM2	5	19	53	112	67
CM3	5	5	35	135	76
CM4	3	6	45	122	80
PEOU1	5	3	45	151	52
PEOU2	4	5	62	137	48
PEOU3	5	3	38	146	64
PEOU4	4	9	58	134	51
TSO1	6	9	46	126	69
TSO2	9	21	67	116	43
TSO3	6	14	65	127	44
TSO4	6	12	63	119	56
PSO1	17	34	73	96	36
PSO2	11	20	71	103	51
PSO3	6	19	77	117	37
PSO4	14	41	88*	78	35
ATT1	3	6	85	121	41
ATT2	3	6	60	146	41
ATT3	5	7	82	132	30
BI1	5	3	73	132	43
BI2	4	7	63	141	41
BI3	7	6	73	126	44
CI1	4	13	67	126	46
CI2	3	14	59	137	43
CI3	5	12	60	133	46

**Table 4.** Mean and standard deviation of the variables.

	Mean	SD
CM	3.98	0.72
PEOU	3.92	0.71
TSO	3.78	0.79
PSO	3.49	0.91
ATT	3.76	0.73
BI	3.79	0.76
CI	3.79	0.82

Table 5 shows the ratio comparison of the dining preference among the OFDS users based on age, gender, marital status and personal income level. Personal income levels were classified as B40 (less than RM4,850), M40 (RM4,851–RM10,970), and T20 (above RM10,971). As expected, the majority of OFDS users preferred to enjoy their food at home during pandemic especially the elderly or married adults prefer to enjoy their food at home (>80% for age group above 41 years old; married 73%). Although 71.88% of the users were earning a low income, they still preferred to use OFDS and dine at home (71%) compared to higher income respondents. This indicates COVID-19 pandemic has significantly changed people’s lifestyles and has became a new norm.

**Table 5.** Comparison of dining preference among the OFDS users.

Characteristic	Ratio	
	Number of OFDS users	Dining at home
**Age**		
<18	4	0.50
18 ~ 25	104	0.68
26 ~ 30	40	0.58
31 ~ 40	71	0.68
41 ~ 50	26	0.81
51 ~ 60	10	1.00
>60	1	1.00
**Gender**		
Female	174	0.73
Male	82	0.60
**Marital status**		
Single	179	0.66
Married	73	0.73
Others	4	1.00
**Personal income level**		
B40	184	0.71
M40	65	0.63
T20	7	0.57

## Measurement of model

### Reliability and validity

Table 6 shows Cronbach’s alpha ^61^^,^^62^ and composite reliability (CR) ^55^^,^^63^^,^^64^ for each variable as above 0.8, which indicates good internal consistency of the questionnaire’s questions scale in measuring a similar variable. * indicates CR > 0.95 but there are no significant changes after its removal. ^55^ The average variance extracted (AVE) indices ^65^ are greater than 0.5 for each variable, indicating no convergent validity problems.

**Table 6.** Cronbach’s alpha, composite reliability and average variance extracted.

	Cronbach’s alpha	Composite reliability	AVE	Item
CM	0.838	0.839	0.566	4
PEOU	0.916	0.916	0.732	4
TSO	0.883	0.883	0.654	4
PSO	0.911	0.911	0.718	4
ATT	0.926	0.927	0.809	3
BI	0.920	0.920	0.793	3
CI	0.964	0.964 *	0.899	3

* Indicates CR > 0.95.

In Table 7 Fornell-Larcker criterion, ^65^^,^^66^ the diagonals represent the square root of AVE and off diagonals represent the coefficient of correlation. One tail t-test is conducted on the coefficient of correlation at 5% level of significance. The results revealed that there is a positive correlation between the variables with *p*-value of 0. There are no discriminant validity issues with the support of HTMT values, recorded in Table 8 based on HTMT _0.90_ criterions. ^67^

**Table 7.** Fornell-Larcker criterion.

	CM	PEOU	TSO	PSO	ATT	BI	CI
**CM**	0.752						
**PEOU**	0.737	0.855					
**TSO**	0.699	0.661	0.809				
**PSO**	0.522	0.534	0.644	0.847			
**ATT**	0.730	0.610	0.677	0.577	0.899		
**BI**	0.758	0.615	0.678	0.542	0.859	0.891	
**CI**	0.607	0.587	0.675	0.565	0.763	0.821	0.948

**Table 8.** Heterotrait-Monotrait ratio (HTMT).

	CM	PEOU	TSO	PSO	ATT	BI	CI
**CM**							
**PEOU**	0.737						
**TSO**	0.698	0.661					
**PSO**	0.542	0.534	0.646				
**ATT**	0.732	0.609	0.676	0.576			
**BI**	0.757	0.615	0.677	0.542	0.859		
**CI**	0.606	0.587	0.674	0.565	0.763	0.821	

### Consistent partial least square (PLSc) path modelling approach

Six hypotheses were tested using PLSc, ^57^ a variance-based structural equation modelling technique, with no concerns about distribution or multicollinearity. In the past decade, the use of PLS modelling has gradually increased in order to handle more complex models.

**Figure 3.**
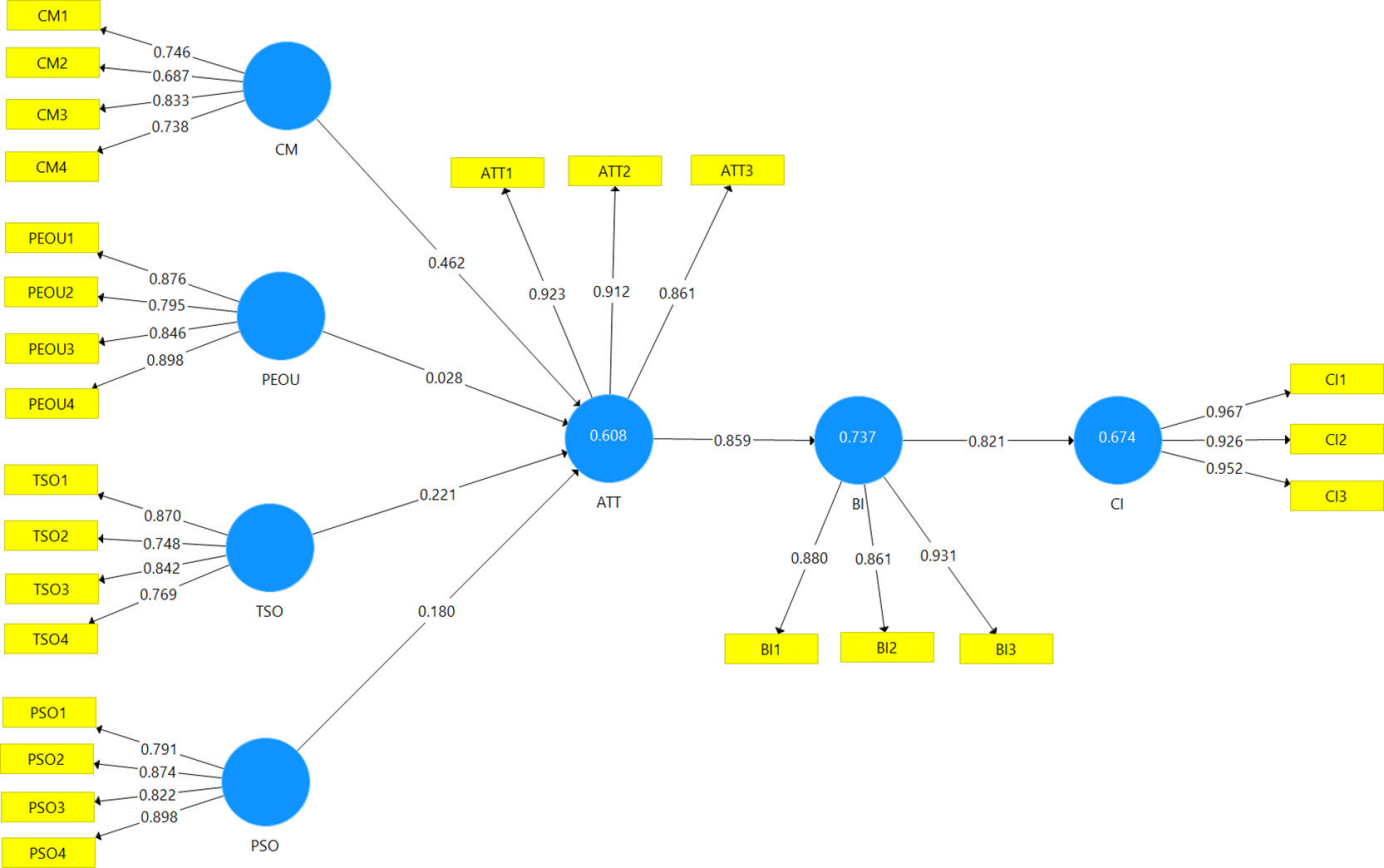
Part coefficient and outer loading.

Table 9 summarises the result of the hypotheses presented in Figure 3, which indicates the path coefficient and outer loading of the variable. PEOU is found to be insignificant in influencing consumers’ attitude towards OFDS ( *p*-value > 0.05). Consumers’ attitude towards using OFDS during and post the COVID-19 pandemic is, however, positively influenced by CM ( *p*-value < 0.05), TSO ( *p*-value < 0.05) and PSO ( *p*-value < 0.05). Furthermore, hypotheses of ATT positively influencing consumers’ BI ( *p*-value < 0.05) and also BI positively influencing consumers’ CI ( *p*-value < 0.05) towards OFDS are supported in this study. Thus, H1, H3, H4, H5 and H6 are validated while H2 is rejected.

**Table 9.** Summary of hypotheses testing.

Hypothesis	Path	t-value	*p*-value	Decisions	f ^**2**^	Q ^**2**^
H1	CM-->ATT	4.119	0.000	Supported	0.134	0.449
H2	PEOU-->ATT	0.287	0.774	Rejected	0.012	
H3	TSO-->ATT	2.187	0.029	Supported	0.055	
H4	PSO-->ATT	2.370	0.018	Supported	0.046	
H5	ATT-->BI	26.390	0.000	Supported	1.706	0.536
H6	BI-->CI	23.985	0.000	Supported	1.493	0.555

To test the model quality, effect size, f ^2^ and predictive relevance, Q ^2^ is measured. All the f ^2^ values are greater than 0.02 except the path of PEOU→ATT, which indicates no effect toward ATT. ^68^^,^^69^ The predictive relevance, Q ^2^ is used to determine the predictive power of dependent variables. All the Q ^2^ values are greater than 0.35. ^70^ This means there is substantial predictive relevance in this model.

## Discussion

Based on the findings of this study, convenience motivation has a significant impact on consumers’ attitude towards OFDS, which is consistent with previous studies. ^10^^,^^11^^,^^18^^,^^22^^,^^33^^,^^35^^–^^37^ OFDS platforms are very well developed nowadays, enabling consumers to order food online at any time and from any location, without having to leave home. With just a click and via a cashless payment system, food will be ready in a short period of time, providing consumers with a great deal of convenience. However, electronic devices have already been integrated into our daily routines for a long time and people are already familiar with these devices, thus perceived ease of use is no longer a significant motivator that would influence consumers to continue ordering food online. ^12^^,^^17^^,^^33^^,^^71^ Instead, usability has become a baseline expectation, preventing dissatisfaction but not actively driving continuance.

Time efficiency is an important factor that consumers, particularly working adults and students, are concerned about. ^10^^,^^20^^,^^33^ Consumers are eager to use OFDS because they can save a significant amount of time from menu selection to food preparation. Especially during rush hour, OFDS will be their first choice rather than waiting in line at a restaurant. Price-saving orientation also influences behaviour, as consumers frequently compare prices across different retailers and look for discounts or take advantage of free delivery, which can encourage repeat usage. Together, convenience, time efficiency, and perceived value appear to be the main factors sustaining post-pandemic OFDS usage.

Previous studies conducted in this field of study have focused on the general intention of using OFDS. ^14^^,^^18^^,^^71^ This paper, however, investigates consumers’ attitude and behaviour regarding their continuance intention of using OFDS after the COVID-19 pandemic. The left-skewed distribution of continuance intention’s measurement items significantly indicates that there is a high possibility of consumers using OFDS continuously after COVID-19, and this supports the hypothesis that a positive behavioural intention will lead to continuance of using a service. A satisfying online shopping experience fosters a positive attitude toward using the services and, as a result, always increases the likelihood of future purchase behaviour. ^21^^,^^72^^,^^73^

Furthermore, many previous studies have integrated TPB & TAM across various contexts, whether it is to investigate mobile banking adoption among Palestinian customers, ^74^ consumer’s willingness to adopt online food in Italy, ^18^ university students’ intention to use mobile learning in Ghana and Colombia, ^75^^,^^76^ Indian commuters’ willingness to use carsharing app ^77^ Indonesians’ intention to use bicycles, ^78^ changes in behaviour of e-wallet users during the COVID-19 pandemic among Indonesians ^79^ or Vietnamese consumers’ online purchase intention ^80^ to name a few. However, to the best of the authors’ knowledge at the time of writing, very few or no studies have included the convenience factor.

The model developed in this study builds on prior TAM and TPB research while incorporating convenience motivation, time-saving orientation, and price-saving orientation to reflect the practical and economic priorities of today’s consumers. These factors help explain the “post-pandemic shift,” showing how OFDS use evolved from a temporary, emergency-response behaviour to a consistent part of everyday life. By capturing these motivational drivers, the framework provides a more nuanced understanding of consumer behaviour and the underlying reasons for continued OFDS usage, especially after the unprecedented pandemic.

The findings have several practical implications for stakeholders. Although perceived ease of use remains important to prevent user dissatisfaction, platform providers and food retailers should focus on enhancing overall convenience, speed, reliability, and perceived value. Platforms can achieve this through features such as fast and reliable delivery, real-time tracking, personalised recommendations, integrated loyalty programmes, and transparent pricing. Food retailers can optimise menu offerings, introduce value-for-money bundles, streamline preparation processes, and adjust pricing or promotions according to peak demand to encourage repeat purchases. Government and policymakers can further support the ecosystem by promoting digital literacy, improving digital payment infrastructure, ensuring fair commission structures, and protecting gig-economy delivery workers to enhance service reliability and inclusiveness. These measures collectively ensure that OFDS platforms provide value not only to consumers but also to small and micro food businesses.

Finally, the study highlights opportunities to reach bottom-of-the-pyramid (BOP) consumers. Convenience, time efficiency, and price savings are particularly important for low-income and underserved populations. Providers can increase accessibility by offering low-cost meal bundles, shared delivery options, subscription-based discounts, and simplified interfaces. Additional measures such as tailored promotions, flexible payment options, and partnerships with local vendors can further expand access while maintaining profitability. By adapting services to meet the needs of price-sensitive consumers, OFDS providers can simultaneously broaden market reach, promote economic inclusion, and sustain business growth, aligning with the notion that businesses can profit while serving underserved populations.

### Limitations

This study did not take into account all of the possible factors that might influence the continuance intention of using OFDS after the pandemic. The model could be improved in the future by including more variables, such as, customer satisfaction and social influences. Furthermore, the findings cannot be generalised as a whole due to convenience sampling biasness. In the future, the study could be narrowed down to a specific group; perhaps looking at some larger cities with higher demand and supply for OFDS.

## Conclusions

OFDS is a consumer-focused market which aims to bring comfort to consumers so that they are able to get their favourite food at the best price and convenience without having to leave home. This is consistent with our findings that convenience motivation, time-saving orientation and price-saving orientation were the primary factors influencing consumers’ attitude towards OFDS during and post the COVID-19 pandemic. The findings also revealed that consumers who have a positive attitude and behaviour towards OFDS tend to have favourable feedback on the continuance intention after COVID-19.

Nevertheless, although results showed that there is a significant impact on the continuance intention towards OFDS after COVID-19, there are several issues and challenges that need to be addressed. Food retailers should consider how to retain the food quality and ensure fast delivery when orders increase. They should also look into collaboration with third-party apps such as GrabFood and Foodpanda to help boost their sales and maximise profits. We believe that consumers will soon adopt OFDS into their lifestyle, making it a norm, after the pandemic. Therefore, it is crucial for food retailers to work in this direction to sustain and grow their business model.

## Data availability

### Underlying data

Figshare: Online Food Delivery Service.

DOI: http://doi.org/10.6084/m9.figshare.14772951. ^59^

This project contains the following underlying data:

• Data file 1. (Survey results, CVS format)

### Extended data

Figshare: Online Food Delivery Service Questionnaire 2021

DOI: http://doi.org/10.6084/m9.figshare.16566414. ^81^

This project contains the following extended data:

• Data file 1. (Survey questions, CVS format)

Data are available under the terms of the Creative Commons Zero “No rights reserved” data waiver (CC0 1.0 Public domain dedication).
