# Supplementary material for: Online food delivery services: cross-sectional study of consumers’ attitude in Malaysia during and after the COVID-19 pandemic
Source: F1000Res. 2026 Mar 3;10:972. Originally published 2021 Sep 27. [Version 3] doi: 10.12688/f1000research.73014.3 (PMC13273245; doi:10.12688/f1000research.73014.3)
Supplement: Peer review history

# Reviewer report: Reviewer response for version 3

**DOI:** 10.5256/f1000research.196577.r486731

**Published:** 2026-06-16

**Authors:** Flavio Santino Bizarrias

**Affiliations:** 1 Escola Superior de Propaganda e Marketing (ESPM), Sao Paulo, Brazil

Dear authors, thank you very much for choosing F1000 Research as the vehicle for disseminating your study, which has provided me with an excellent learning opportunity.

 I present the following comments to contribute to the indexing of your study.

 - Positive points of the study

 The manuscript presents a series of relevant positive points that the authors suggest as their academic and managerial contribution to the consumer behaviour literature. Initially, the work suggests theoretical and methodological robustness by integrating the Technology Acceptance Model and the Theory of Planned Behaviour, which constitute two of the most consolidated and widely validated conceptual frameworks in the scientific literature for explaining the acceptance and use of information systems. This choice provides a solid framework for the development of both the measurement and structural models. On the empirical level, the validation of the data collection instrument revealed reliability and convergent validity indicators, with Cronbach's alpha and Composite Reliability exceeding the standard threshold across all studied constructs, suggesting internal consistency of the adapted scales. Similarly, the Average Variance Extracted indices fell entirely above the recommended threshold, ensuring that the model adequately captures data variability. Regarding the evaluation of the structural model through structural equation modelling, the empirical results demonstrated explanatory power and statistical stability. The model achieved adequate coefficients of determination and substantial predictive relevance indices, all established above the limit proposed in the literature, which indicates the adequacy of the independent variables in anticipating consumer attitudes and intentions. Furthermore, effect sizes proved to be significant in the main causal relationships tested, reinforcing the real impact of utilitarian constructs on the continuance intention to use delivery services. From a managerial and social perspective, the article suggests moving forward by extending its practical implications to the bottom of the pyramid consumer segment. The authors detail how factors linked to convenience, time saving and price optimisation align directly with the needs of lower-income populations and local small businesses, providing valuable insights for service providers to develop inclusive and accessible offerings. This practical focus demonstrates how the benefits of digital platforms can be directed to promote economic inclusion and market growth in a sustainable manner. The study suggests fulfilling an essential pedagogical role in the debate on digital maturity by empirically confirming the rejection of the direct impact of perceived ease of use on post-crisis attitudes. This signals to the market that simple usability has ceased to be a competitive advantage generating active satisfaction and has become a basic technological hygiene expectation, forcing organisations to seek differentiation through real convenience proposals and economic value generation.

 - Points to develop

 Regarding the introduction and macroeconomic contextualisation, the article demonstrates soundness by presenting a robust overview filled with statistical data on the growth of the delivery sector during the peak of the global health crisis, citing the advancement of market revenues and the expansion of major platforms such as GrabFood and Foodpanda. However, this density of contextual data ends up eclipsing the clear delimitation of the research problem, causing the knowledge gap to be introduced in an overly timid and peripheral manner. The text dedicates a disproportionate amount of space to describing the empirical reality of the post-pandemic period, generating a structural imbalance that weakens the justification of the scientific study. The central argument relies on the premise of understanding whether the surge in consumption was a purely temporary phenomenon or if it would result in sustained long-term growth. This approach lacks depth, as the authors only superficially mention that previous investigations focused on general attitudes towards online services and that few researchers focused on specific experiences with meal delivery platforms. Although the following sections enumerate various isolated attributes addressed by contemporary literature, the manuscript fails to specify precisely the theoretical advancement that its proposal offers beyond what is already consolidated. The mere justification of investigating continuance intention in a post-pandemic scenario loses argumentative force at the current moment, given that the end of mobility restrictions and the return to social normalcy have already been temporally consolidated in global daily life. The theoretical foundation is built upon the integration of the Technology Acceptance Model and the Theory of Planned Behaviour, which are two classic and widely validated approaches in the information systems and consumption literature. While the adoption of these established frameworks lends legitimacy and conceptual support to the proposed model on the one hand, this choice restricts the innovative character and originality of the work on the other, a dilemma that requires a more robust critical discussion throughout the text. The authors seek to extend these traditional models by incorporating the variables of convenience motivation, time-saving orientation and price-saving orientation. However, there is a lack of a robust conceptual justification explaining in detail the psychological mechanisms behind the inclusion of these specific dimensions and how they expand the frontier of existing knowledge on the subject. The organisation of the text incurs a stylistic and structural redundancy problem there in the introduction, since the explanation of these new theoretical constructs and their relevance to the period following the health crisis are repeated in an almost identical manner in different parts of the literature review. Another critical point in building the framework is the absence of a logical and individualised sequence for formulating the research hypotheses. By presenting all supporting theoretical arguments in a single block and listing the set of hypotheses only at the end of the section, the authors compromise methodological clarity, preventing the reader from understanding the exact moment when each construct articulates to justify a specific causal relationship. The structure of the structural model itself evokes conceptual questions that were not properly addressed within the scope of the article. The proposed linear configuration establishes that attitude influences behavioural intention, which in turn directly impacts continuance intention. This modelling ignores the possibility of behavioural intention acting as a full or partial mediating variable in the relationship between attitude and continuance intention, an important theoretical omission that lacks a well-founded justification in the marketing literature. Regarding the described methodological procedures, the method section is excessively succinct and lacks depth when discussing the specificities of the chosen statistical techniques. Although the authors mention using the Consistent Partial Least Squares approach and computerised bootstrap resampling routines with five thousand subsamples through the SmartPLS software, they do not deepen the discussion regarding the operational assumptions of variance-based structural equation modelling, nor do they justify the adequacy of this technique in relation to covariance-based methods, which were neglected in the study. This methodological fragility extends to the presentation and pre-treatment phase of the quantitative data obtained in the research. The reporting of the results jumps directly into the description of the demographic profile and the statistical testing of the hypotheses without making any mention of fundamental data exploration and screening procedures. The article completely omits the performance of tests to detect outliers that could distort the estimates, fails to address the verification of common method bias resulting from self-reported data collection at a single point in time, and neglects the formal diagnosis of multicollinearity among the independent predictor variables. From a strictly theoretical perspective, the final model tested confirms the rejection of the hypothesis linking perceived ease of use to consumer attitude, demonstrating that the addition of traditional utilitarian variables adds little incremental value to theory in a digital environment that is already mature and widely dominated by users. Beyond the standard path coefficients and the effect and predictive relevance indices reported in the descriptive table, the manuscript ignores the most recent methodological recommendations in the econometrics literature that advocate the systematic inclusion of confidence intervals for all obtained structural estimates. A severe construct validity problem manifests itself explicitly in the analysis of the variable metric differentiation criteria table. A meticulous examination of the matrix based on the Fornell-Larcker criterion reveals a discriminant validity inconsistency that was completely ignored or omitted in the discussion of the results. The square root of the average variance extracted for the convenience motivation construct presents a value of 0.72, an index that is numerically lower than the linear correlation observed between this same construct and the behavioural intention variable, which reaches the 0.758 mark, suggesting at the very least an equivalence between these constructs. According to established statistical precepts, the internal shared variance of any latent variable must systematically be higher than the variance shared with the other factors in the model, meaning that the observed behaviour breaks this rule and indicates that the convenience and behavioural intention constructs may be capturing the same empirical phenomenon, thereby compromising the purity of the statistical conclusions. Finally, the temporal distance of the study emerges as one of the most evident and challenging limitations to the scientific relevance of the manuscript. The primary data sustaining the entire quantitative analysis were collected between March and April of 2021, placing the research in a distant past and within a social context radically different from the current one. This substantial chronological distance demands a much more robust and profound argumentative effort from the authors than what is presented in the current version. It is imperative to expand the theoretical discussion section to justify how the results gathered in 2021 can be interpreted and applied to the contemporary reality of the consumer market, transforming what appears to be a temporal anachronism into a valuable historical contribution. The manuscript needs to demonstrate clearly whether the attitudes mapped at the height of social isolation have indeed consolidated into a stable pattern of routine behaviour in today's society or if consumer priorities have undergone new structural mutations, thus allowing the study to serve as a longitudinal anchor point to understand the evolution of digital consumption in moments of pandemic and macroeconomic stability.

 Best regards,

 The reviewer

Is the work clearly and accurately presented and does it cite the current literature?

Yes

If applicable, is the statistical analysis and its interpretation appropriate?

Partly

Are all the source data underlying the results available to ensure full reproducibility?

Yes

Is the study design appropriate and is the work technically sound?

Partly

Are the conclusions drawn adequately supported by the results?

Yes

Are sufficient details of methods and analysis provided to allow replication by others?

Partly

Reviewer Expertise:

Expert in consumer behaviour and quantitative methods.

I confirm that I have read this submission and believe that I have an appropriate level of expertise to confirm that it is of an acceptable scientific standard, however I have significant reservations, as outlined above.

# Reviewer report: Reviewer response for version 2

**DOI:** 10.5256/f1000research.165223.r283065

**Published:** 2024-07-11

**Authors:** Lau Teck Chai

**Affiliations:** 1 Entrepreneurship and Enterprise Hub, Xi'an Jiaotong-Liverpool University, Suzhou, China

The version of the article has gone through revision based on the review from 2 previous reviewers. The authors have addressed all the major concerns pointed out earlier. However, there are some concerns that I would like to highlight (below) for further improvement.

 1. Abstract - Under the Method section, the authors still did not change the data collection method (From convenience to purposive?)

 2. Page 4 - *"This comprehensive framework combines elements from the TAM and the TPB, augmenting them with additional variables"* However, in the study's research model, some constructs were omitted (subjective norm and perceived behavioral control). The authors did not explained the reason(s) for the exclusion of the two constructs.

 3. Furthermore, how do the new added constructs (convenience motivation, time saving orientation, price saving orientation) are decided upon to be included in the TAM+TPB model? Are the new constructs adopted from any established theoretical model? Any particular reason(s) for their inclusion?

 4. Page 6 - Figure 1 Research Model Diagram - Attitude is to be represented by ATT and not AI on the diagram.

 5. Page 6 - *All items were measured based on a*

*five-point Likert-type.* Please specify how they are measured specifically.

 6. Page 6 - *In this study, purposive sampling method was applied because the selected samples are more representative of the population.* In what way are they more representative? Most likely because respondents need to have experience in online food ordering? Do you have screening questionnaire?

 7. Page 6 - The authors will need to explained that only those who have experience of purchasing online will be qualified to answer the survey. 

 8. Page 6 - *All of them has experienced using OFDS and mostly are young adults between the age of 18 to 25 years old (40.63%).* This criteria need to be explained earlier during the research design stage.

 9. Page 7 - A *lthough 71.88% of the users were earning a low income.*  It is unclear on the low income group and how much they earned. The Table only mentioned classification (B40). This classification need to be explained for non-Malaysian readers who are unfamiliar with such classification.

 10. Page 10 - *electronic devices have already been*

*integrated into our daily routines for a long time and people are already familiar with these devices, thus perceived ease of use is not a significant motivator that would influence consumers to continue ordering food online. *Please give suggestions to companies, and platform providers on what this means to them and what they need to do and be aware.

 11. Page 11 - *but perhaps not for all students.* I am not sure about the relevance of this sentence, as the sample consists of respondents who are not only students. 

 12. Page 12 - *It is a valuable framework for understanding consumer behaviour, product design, and service delivery in a wide range of contexts, from OFDS to technology adoption and beyond, especially after the unprecedented pandemic. *What are some tangible suggestions for food retailers, platform providers, government/policy makers? Perspective and discussion provided are mainly from consumers perspective. 

 13. Following up on point 12 above, detailed suggestions for businesses and retailers would be appropriate as it will aligned with what the authors have mentioned in their introduction section (page 3), quoted below:

*If businesses, especially, could modify their business models to cater to the population at the bottom of the pyramid, they could play a significant role in alleviating*

*poverty while still profiting.*

*This would assist food retailers in positioning their products and services to capitalise on this emerging market.*

Is the work clearly and accurately presented and does it cite the current literature?

Yes

If applicable, is the statistical analysis and its interpretation appropriate?

Yes

Are all the source data underlying the results available to ensure full reproducibility?

Yes

Is the study design appropriate and is the work technically sound?

Yes

Are the conclusions drawn adequately supported by the results?

Yes

Are sufficient details of methods and analysis provided to allow replication by others?

Yes

Reviewer Expertise:

Marketing, Consumer Behaviour

I confirm that I have read this submission and believe that I have an appropriate level of expertise to confirm that it is of an acceptable scientific standard, however I have significant reservations, as outlined above.

# Response

**Published:** 2026-02-12

**Authors:** Sin Yin Tan

**Affiliations:** Multimedia University, Malaysia

Thank you so much for your comments. We sincerely appreciate the valuable feedback and suggestions from you.

 To address the comments that highlighted by reviewer:

 1.We would like to apologise for overlooking this when revising the manuscript previously. It has been amended accordingly in the Abstract section.

 2.Although subjective norm (SN) and perceived behavioural control (PBC) are key components of the Theory of Planned Behaviour, their influence varies depending on the stage of technology use. Our study focuses on the continuance stage of OFDS, where users are already familiar with the platform. In this context, PBC is less influential because users already have the necessary skills and resources, and SN is less relevant as ordering food online is typically a private, routine behaviour. At this stage, continued use is primarily shaped by users’ evaluations of usefulness, convenience, and value rather than social pressure or perceived control. We have clarified this reasoning and strengthened the explanation in the revised Theoretical Background section to justify the model specification.

 3.We have added Convenience motivation (CM), time-saving orientation (TSO), and price-saving orientation (PSO) into the model to better reflect today’s realities. The original TPB is great for explaining why someone starts using a technology, but we are moving beyond the broad technology adoption perspective and looking at the actual utilitarian and economic motivations that define routine OFDS use today. We have clarified this theoretical refinement in the revised manuscript to ensure the model reflects both established behavioural theory and the practical motivations of users in a post-pandemic OFDS environment.

 4.“ATT” has been inserted accordingly in Figure 1.

 5.We have added the clarification for the five-point Likert scale in the Questionnaire Development section.

 6.The survey contains a screening question to ensure only respondents that have experience using online food delivery services proceed with the questionnaire. We have added a statement under the Data collection section.

 7.Yes, this has been addressed in No. 6 – We have added a statement under Data Collection section to indicate that there is a screening question to ensure those who answered the questionnaire has experience using OFDS.

 8.Already addressed in No. 6 - We have added a statement under Data Collection section to indicate that there is a screening question to ensure those who answered the questionnaire has experience1e have added a sentence under Results to indicate how B40, M540 and T20 are classified.

 9.Thank you for pointing this out. We have added a sentence under Results to indicate how B40, M540 and T20 are classified.

 10.Regarding perceived ease of use, our findings show it does not significantly drive continuance intention. This reflects that usability has become a baseline expectation in mature digital environments like OFDS. Consumers are already familiar with smartphones, apps, and digital payments, so ease of use now primarily prevents dissatisfaction rather than actively motivating continued use. We have clarified in the revised Discussion that platform providers should maintain stable, intuitive, and reliable interfaces while focusing differentiation on enhancing convenience and value, such as faster delivery, accurate orders, personalised recommendations, and seamless integration with payment and loyalty systems. This explanation has been added to provide practical guidance for stakeholders in post-pandemic OFDS markets.

 11.We have removed the sentence to avoid confusion.

 12.We agree that the findings should provide clearer practical implications. In the revised Discussion section, we have added a brief paragraph outlining how the results can inform platform providers, food retailers, and policymakers.

 13.We thank the reviewer for highlighting the importance of bottom-of-the-pyramid consumers. In response, the Discussion now explicitly addresses strategies to reach this segment.

# Reviewer report: Reviewer response for version 2

**DOI:** 10.5256/f1000research.165223.r275580

**Published:** 2024-06-08

**Authors:** Hyun-Woo Joung

**Affiliations:** 1 The University of Mississippi, Lubbock, USA

The authors have addressed the previous comments, and the current revised manuscript meets the necessary standards. The manuscript is now acceptable for indexing.

 Thank you for your efforts in making the required revisions.

Is the work clearly and accurately presented and does it cite the current literature?

Partly

If applicable, is the statistical analysis and its interpretation appropriate?

Yes

Are all the source data underlying the results available to ensure full reproducibility?

Yes

Is the study design appropriate and is the work technically sound?

Yes

Are the conclusions drawn adequately supported by the results?

Partly

Are sufficient details of methods and analysis provided to allow replication by others?

Partly

Reviewer Expertise:

Consumer behavior in the hospitality industry

I confirm that I have read this submission and believe that I have an appropriate level of expertise to confirm that it is of an acceptable scientific standard.

# Response

**Published:** 2024-06-28

**Authors:** Sin Yin Tan

**Affiliations:** Multimedia University, Malaysia

Thanks again for your feedbacks.

# Reviewer report: Reviewer response for version 1

**DOI:** 10.5256/f1000research.76632.r258703

**Published:** 2024-05-06

**Authors:** Hyun-Woo Joung

**Affiliations:** 1 The University of Mississippi, Lubbock, USA

Introduction:

- Strengthen the introduction by providing more rationale for the study and identifying research gaps.
- Clarify whether the study was conducted recently or in 2021, and update statistics accordingly.
- Address the discrepancy between data collection during the pandemic in 2021 and discussing the impact "after" the pandemic.

Literature Review:

- Expand the literature review to provide more in-depth analysis of potential predictor variables or offer detailed reasoning for the chosen variables.
- Strengthen the hypotheses development section by providing clear rationale for each hypothesis.
- Consider adopting a relevant theory (e.g., UTAUT, TAM) to support the conceptual model.

Methods:

- Provide a brief explanation of the Krejcie and Morgan sampling method to enhance understanding.

Results:

- Address ethical concerns regarding participants under the age of 18 and clarify how their data were handled.
- Explain how participants who have not used OFDS before were able to answer questions related to "Continuance intention."
- Evaluate the necessity of Table 3 and consider its relevance to the study.
- Enhance Table 5 by providing additional statistical evidence, such as chi-square analysis, in addition to ratios.
- Explain the discrepancy in Table 7 where CM's square root of AVE is smaller than the correlation between CM and PEOU.

Discussion and Conclusion:

- Offer a more in-depth discussion that goes beyond repeating the findings already discussed in the results section.
- Strengthen the practical and theoretical implications.

Overall:

- Acknowledge the importance of the topic in the restaurant industry.
- Suggest improvements to enhance the quality of the manuscript, including professional proofreading.

Is the work clearly and accurately presented and does it cite the current literature?

Partly

If applicable, is the statistical analysis and its interpretation appropriate?

Yes

Are all the source data underlying the results available to ensure full reproducibility?

Yes

Is the study design appropriate and is the work technically sound?

Yes

Are the conclusions drawn adequately supported by the results?

Partly

Are sufficient details of methods and analysis provided to allow replication by others?

Partly

Reviewer Expertise:

Consumer behavior in the hospitality industry

I confirm that I have read this submission and believe that I have an appropriate level of expertise to confirm that it is of an acceptable scientific standard, however I have significant reservations, as outlined above.

# Response

**Published:** 2024-05-15

**Authors:** Sin Yin Tan

**Affiliations:** Multimedia University, Malaysia

Thank you so much for your comments. We sincerely appreciate the valuable feedback and suggestions from you.

 To address the comments that highlighted by reviewer:

 1.Introduction:

- The research gaps and contributions of our study have been restructured to include the justification for the research under the introduction section. The study was conducted in 2021 as stated in our article. In future, we plan to extend our study on the impact “after” the pandemic in the OFDS domain.

2.Literature Review:

- Theoretical Framework has been included under the Literature Review section. Our selected variables were based on the Theory of Planned Behavior (TPB) and the Technology Acceptance Model (TAM). Detailed elaboration was further described in this section.

3.Methods:

- Purposive sampling method was applied in our study and clarified under Data collection of the Methods section. The sampling size was based on the other studies in the same domain.

4.Results:

- Research ethics approval was obtained from Multimedia University, Malaysia (EA1422021) and the respondents gave their written informed consent when filling out the Google Form.
- The survey data underwent reanalysis by excluding those who did not have any experience using Online Food Delivery Services (OFDS). Therefore, the new demographic profile has been presented under the Result section.
- Table 3 illustrates feedback of respondents for each measurement item. The objective is to show that there is a significance left-skewed for each measurement item except PSO4.
- In this study, descriptive study is maybe more meaningful for certain variables such as the mature adults (above 41 years old), and B40 respondents (personal Income level) preferred to dine at home. The preference of dining at home is analysed based on their demographic so that sellers can approach these groups of people.
- In Table 7, CM's square root of AVE is greater than the correlation between CM and PEOU.

5.Discussion and Conclusion:

- Discussion and conclusion section have been further improved by explaining how this study contribute to existing theoretical frameworks based on the proposed research model. Further descriptions of related works in this domain were also provided.

6.Overall:

- Thank you for your suggestion. A significant revision had been undertaken which involves restructuring of the research study based on the theoretical framework and subsequent analysis.

# Reviewer report: Reviewer response for version 1

**DOI:** 10.5256/f1000research.76632.r100846

**Published:** 2021-12-21

**Authors:** Bui Thanh Khoa

**Affiliations:** 1 Industrial University of Ho Chi Minh City, Ho Chi Minh City, Vietnam

Thank you very much for the opportunity to review the study titled “Online food delivery services: cross-sectional study of consumers’ attitude in Malaysia during and after the COVID-19 pandemic.” The selected topic is intriguing, and the work can add value to the existing body of literature. However, certain flaws overshadow the paper’s potential significance. In the following lines, I offer some suggestions to this study as follows:

1. One of the most pressing challenges is the theoretical research gap. Please provide a well-defined research gap. Furthermore, please explain why it is critical to include comprehensive poverty eradication in the theoretical contribution for the study area; hence, restructure the introduction. Typically, the framework will include the following elements: the significance of the issue, motivation (optional), research gap(s), aims, and possible contributions (optional). Lack of the research gap(s) reduces the paper’s value.
2. This research used many constructs, such as Convenience motivation, Perceived ease of use, Time-saving orientation,  Price-saving orientation, Attitude, Behavioural intention, and continuance intention; however, the reviewer cannot find the theoretical background in this research to connect them; hence, the proposed model is subjective and lacks scientific arguments. Please add the theoretical background in the first literature review.
3. Table 2 shows the demographic profile of 307 respondents, of which there are 16.61% of respondents that have not used OFDS before. Why can they answer the research items regarding the continuance intention?
4. The sampling method should be based on the research objective more than the prior research.
5. The data analysis should add the f ^2^ and Q ^2^ values to check the effect size and predictive relevance.
6. Limitation should be moved to the end of the paper.
7. I firmly believe that the authors need a significant overhaul of the study, especially the theoretical framework and data process for ensuring the scientific validity of the article to make the manuscript suitable for indexing, given that it will be professionally revised before another submission.

Is the work clearly and accurately presented and does it cite the current literature?

Yes

If applicable, is the statistical analysis and its interpretation appropriate?

Yes

Are all the source data underlying the results available to ensure full reproducibility?

Partly

Is the study design appropriate and is the work technically sound?

No

Are the conclusions drawn adequately supported by the results?

Partly

Are sufficient details of methods and analysis provided to allow replication by others?

Partly

Reviewer Expertise:

electronic commerce, online consumer behavior, marketing

I confirm that I have read this submission and believe that I have an appropriate level of expertise to state that I do not consider it to be of an acceptable scientific standard, for reasons outlined above.

# Response

**Published:** 2024-04-17

**Authors:** Sin Yin Tan

**Affiliations:** Multimedia University, Malaysia

Thank you so much for the reviewer's comments. We sincerely appreciate the insightful feedback provided by the reviewer.

 To address the comments that highlighted by reviewer:

1. The Introduction section has been substantially revised by including more significance of the research issues and highlighting the research gaps and contributions of our study.
2. The theoretical background is now positioned within the Literature Review section. We elaborate our study based on the Theory of Planned Behavior (TPB) and the Technology Acceptance Model (TAM).
3. The survey data underwent reanalysis by excluding those who did not have any experience of using Online Food Delivery Services (OFDS). Therefore, the new demographic profile has been presented under Result section.
4. Purposive sampling method was applied and clarified under Data collection of the Methods section.
5. As suggested by reviewer, the f ^2^ and Q ^2^ values have been added in Table 9 under Result section to check the effect size and predictive relevance.
6. Considering the journal editor's advice, the limitation was suggested to place before Conclusions section.
7. A significant revision had been undertaken which involve restructuring of the research study based on the theoretical framework and subsequent analysis.

Thank you.
